# Antifungal efficacy and mechanisms of *Bacillus licheniformis* BL06 against *Ceratocystis fimbriata*

**DOI:** 10.3389/fpls.2025.1535296

**Published:** 2025-02-28

**Authors:** Fangyuan Gao, Dongjing Yang, Jingwei Chen, Xiaosi Zhou, Chengling Zhang, Jukui Ma, Wei Tang, Zhao Liang, Yongwang Wu, Hongxia Liu, Houjun Sun

**Affiliations:** ^1^ Xuzhou Institute of Agricultural Sciences in Jiangsu Xuhuai District, Key Laboratory of Biology and Genetic Improvement of Sweet Potato, Xuzhou, Jiangsu, China; ^2^ Jiangsu Coastal Area Institute of Agricultural Sciences, Yancheng, China; ^3^ Suzhou Bainuo Biomedical Technology Co., LTD, Sequencing Department, Suzhou, Jiangsu, China; ^4^ Department of Plant Pathology, College of Plant Protection, Nanjing Agricultural University, Nanjing, China

**Keywords:** *Bacillus licheniformis* BL06, *Ceratocystis fimbriata*, biological control, sweet potato, transcriptome

## Abstract

Sweet potato black rot caused by the pathogenic fungus *Ceratocystis fimbriata* is a destructive disease that can result in severe agricultural losses. This study explores the antifungal efficacy and underlying mechanisms of *Bacillus licheniformis* BL06 against *C. fimbriata*. The plate antagonism assay revealed that BL06 significantly suppressed the radial growth of *C. fimbriata* mycelia, achieving inhibition rates of 39.53%, 53.57%, 64.38%, and 69.11% after 7, 10, 13, and 16 days, respectively. *In vivo* experiments demonstrated that BL06-treated sweet potato tissues exhibited markedly smaller lesions than the control, indicating effective suppression of black rot. Microscopic observations indicated that BL06 treatment altered the morphology and activity of *C. fimbriata* mycelia, causing swelling and deformation. Additionally, BL06 markedly reduced spore production and germination in a dose-dependent manner, with complete inhibition observed at the highest concentrations tested. The cell-free supernatant (CFS) of BL06 was identified as the primary antifungal agent, achieving an inhibition rate of 76.11% on mycelial growth. Transcriptome analysis of *C. fimbriata* treated with BL06 CFS revealed significant downregulation of genes involved in cell wall and membrane biosynthesis, spore development, protein processing in the endoplasmic reticulum, and energy metabolism. These findings suggest that BL06 is a potent biocontrol agent against *C. fimbriata*, exerting its effects through multiple molecular pathways.

## 1 Introduction

Sweet potato (*Ipomoea batatas* (L.) Lam.), a nutrient-rich tuber crop abundant in vitamins, proteins, minerals, dietary fiber, and carotenoids, is a crucial agricultural product used for food, animal feed, and industrial purposes (Behera et al., 2022; Mohanraj and Sivasankar, 2014). Its critical role in global food security and nutrition highlights the importance of sustaining sweet potato production. However, sweet potato is susceptible to be infected by various pathogenic fungi during field, transportation and storage (Chakraborty et al., 2017). Among these diseases, sweet potato black rot, caused by *C. fimbriata*, is one of the most destructive fungal threats to the sweet potato industry. It is not only prevalent worldwide in major sweet potato-producing regions but also causes significant economic and agricultural losses (Stahr and Quesada-Ocampo, 2020; Sun et al., 2020). The disease not only reduces sweet potato yields and quality, leading to significant economic losses, but also triggers the production of fungal toxins like ipomeamaron, thereby posing a considerable risk to the health of both humans and animals (Ray and Ravi, 2005; Zhang Y. J. et al., 2023).

Currently, various of management strategies, including low-temperature storage, breeding resistant varieties and chemical control, have been employed to control sweet potato black rot disease (Liu et al., 2023; Scruggs et al., 2017). However, these methods present specific agricultural practicality issues, such as the high cost of low-temperature storage and the limitations on breeding resistant varieties (Gong et al., 2022; Parada-Rojas et al., 2021; Xing et al., 2018). The application of synthetic chemical fungicides is an effective measure for controlling sweet potato black rot, but it may lead to a series of problems, including environmental pollution and the presence of toxic residues in agricultural products (Liu et al., 2023; Guzmán et al., 2023; Tleuova et al., 2020). Hence, there is an urgent demand for environmentally friendly, practical, and sustainable solutions to manage sweet potato black rot effectively.

Utilizing biological control agents is a promising and eco-friendly alternative that has gained attention in recent years (Akoetey et al., 2017; Tariq et al., 2020). Among them, *Bacillus* species have emerged as particularly effective biocontrol agents due to their adaptability, resilience, and strong antifungal properties. *Bacillus licheniformis*, a gram-positive bacterium known for its resilience and adaptability, shows considerable potential in managing plant diseases (Fira et al., 2018; Muras et al., 2021). For instance, the endophytic bacterium *B. licheniformis* GL74 can inhibit the growth of various plant pathogenic fungi *in vivo* and *in vitro*, and reduce the area of gray mold on grape leaf surface (Nigris et al., 2018). *B. licheniformis* MH48, isolated from rhizosphere soil, exhibits significant control efficacy against rice sheath blight, anthracnose, and *Phytophthora* disease in capsicum, with its filtrate demonstrating strong inhibitory effects on plant pathogenic fungi (Jeong et al., 2017). *B.licheniformis HN-5* produces bacitracin A, which demonstrates strong antibacterial activity against *Pantoea ananatis* by damaging its cell wall and membrane and altering gene expression related to cell division and biosynthesis (Peng et al., 2021). Additionally, the suspension of *B. licheniformis* W10, combined with bacteriostatic proteins, significantly inhibits the growth of *Monilinia fructicola*, demonstrating effective biocontrol for peaches during storage (Ji et al., 2020; Zhaolin et al., 2015). These studies illustrate the considerable potential of *B. licheniformis* as a biocontrol agent in managing various plant diseases.

Nowadays, several microbes have been reported to be effective against *C. fimbriata.* For instance, *Bacillus amyloliquefaciens* XZ-1 can significantly inhibit mycelium growth and spore germination of *C. fimbriata*, and the cell-free supernatant (CFS) can significantly reduce the volume of the spot, with a control effect of 65.02% (Yang et al., 2018). *B. amyloliquefaciens* YTB1407 had a noticeable antifungal effect on the mycelia of *C. fimbriata* and could increase the salicylic acid content of sweet potato (Wang et al., 2020). Volatile organic compounds (VOCs) produced by *Streptomyces setonii* WY228 and *Streptomyces lavendulae* SPS-33 could inhibit the growth of *C. fimbriata* and induce the expression of disease-resistance-related substances to protect sweet potato (Gong et al., 2022; Li et al., 2020). *Bacillus altitudinis* P32-3 and *Streptomyces djakartensis* MEPS155 can inhibit the growth of *C. fimbriata* mycelium both *in vivo* and *in vitro*, and exhibit good biocontrol effect on sweet potato. In addition, the CFS of *B. altitudinis* P32-3 and *S.djakartensis* MEPS15 demonstrate strong inhibitory effects on mycelial growth (Zhang Y. J. et al., 2023; Zhang et al., 2024). However, despite the promising potential of *B. licheniformis*, its application in controlling sweet potato black rot remains underexplored, particularly with regard to its antifungal mechanisms.

Previous studies have shown that strain BL06 effectively inhibits the growth of various plant pathogenic fungi and demonstrates significant biocontrol potential (Li et al., 2020; Liu et al., 2023; Wang et al., 2014). However, the antifungal efficacy of BL06 against *C. fimbriata* and the mechanisms of action are still unknown. Therefore, the aims of this study are (1) to evaluate the antifungal effects of strain BL06 against *C. fimbriata* growth *in vitro*; (2) to assess the protective effects of strain BL06 against *C. fimbriata*; and (3) to explore the possible antifungal mechanism of *C. fimbriata* responds to strain BL06 by transcriptome analysis. The results of this study can provide valuable insights into the biological control of sweet potato black rot.

## 2 Materials and methods

### 2.1 Strains and culture conditions

The *C. fimbriata* BMPZ13 strain was previously isolated from diseased tubers, which were identified by ITS, and stored in the Jiangsu Xuzhou Sweet potato Research Center. The *C. fimbriat*a strain was cultured on potato dextrose agar (PDA) medium (200 g/L potato, 20 g/L dextrose, and 16 g/L agar powder). The BL06 (provided by Dr. Liu Hongxia of the Nanjing Agricultural University) was cultured on Luria Bertani (LB) agar (1% Tryptone, 0.5% Yeast extract, 1% NaCl and 1.5% agar) at 37°C. *Ipomoea batatas* (L.) Lam. cv. Xu shu 18 was used in this study.

### 2.2 Control effect of BL06 against *C. fimbriata* in sweet potato

To assess the biocontrol efficacy of BL06, sweet potato roots, leaves, and stems of the “Xu18” cultivar were inoculated. The leaves and stems (5-6 weeks old) were punctured using sterile syringe needles. Subsequently, 15 μL of BL06 bacterial suspension (1 × 10^7^ CFU/mL) was applied as a pretreatment for one day, with 15 μL of sterilized ddH_2_O serving as the control. Inoculation sites were then treated with 2 μL of *C. fimbriata* spores suspension (1 × 10^6^ spores/mL). Finally, the leaves and stems were covered with plastic wrap and incubated at 28°C for 36 hours. The pathogenicity assay of tuber consisted of four treatments: (i) the tuber was only prick the hole with a needle tip, (ii) tuber inoculated with BL06 suspension(15 µL), (iii) tuber inoculated with *C. fimbriata* spore suspension (2 µl), and (iv) tuber cultured with BL06 suspension (15 µL) 1day later, then inoculated with *C. fimbriata* spores (2 µl). Tuber samples were placed in a sterilized box, moistened with sterilized gauze, and incubated at 28°C. The area of the infected wounds was measured after ten days using ImageJ software. Each group had three replicates, and the experiment was repeated three times.

### 2.3 Detection of the antagonistic effect of strain BL06 on sweet potato black rot

The pellet was resuspended in sterilized water to achieve a concentration of 1 × 10^7^ CFU/mL. Mycelial discs (5 mm in diameter) from actively growing *C. fimbriata* culture were placed centrally on PDA agar plates. Two sterilized round filter papers were symmetrically positioned 2.5 cm from the center of the plates, each receiving 2 µL of the BL06 suspension. Control plates contained only mycelial discs. The plates were sealed and incubated at 28°C. Each treatment was replicated three times, and the experiment was conducted in triplicate. Mycelial growth was measured at various intervals, and the inhibition rate was calculated using the formula:


Inhibition rate(%)=(diameter of the control-diameter of the treatment)/(diameter of the control)× 100

### 2.4 Effect of BL06 on mycelium morphology of *C. fimbriata*

Exactly 100 µL strain BL06 (1 × 10^6^ CFU/mL) bacterial culture was added to a sterilized 250 ml conical flask containing 50 ml PDA liquid medium. An equal volume of LB liquid medium was added to the control. Three 5 mm diameter discs of the *C. fimbriata* were placed in the 250 ml conical flask, and incubated in a constant temperature incubator at 28°C in the dark. Small mycelia samples were taken after 3, 5, and 7 days of incubation, and their morphology was observed under a light microscope. Concurrently, Evans blue (0.5 mg/mL) and neutral red (0.1 mg/mL) staining were used to assess the viability of *C. fimbriata* mycelia, utilizing a previously described staining technique (Jiang et al., 2019).

### 2.5 Effect of BL06 on spore production and germination of *C. fimbriata*

To evaluate the effect of BL06 on spore production, five mycelial plugs (5 mm in diameter) were taken from the actively growing edge of *C. fimbriata* cultures and transferred into Petri dishes (9 cm × 9 cm) containing 20 mL of PDA liquid medium. BL06 bacterial suspension was added to the medium at varying concentrations, while a PDA-only group (without BL06) served as the control. The cultures were incubated at 28°C for 5 days. After incubation, spores were counted in three separate microscopic fields for each replicate. Each treatment was performed in triplicate, and the entire experiment was repeated three times to ensure reproducibility.

Introduce 1 mL of spore suspension into a sterile 2 mL centrifuge tube, then add 1 mL of BL06 bacterial suspension, ensuring thorough mixing. The concentrations of the bacterial suspension were as follows: 1 × 10^7^ CFU/mL, 1 × 10^6^ CFU/mL, 1 × 10^5^ CFU/mL, 1 × 10^4^ CFU/mL, 1 × 10^3^ CFU/mL, 1 × 10^2^ CFU/mL. PDA and water groups served as controls. The spore suspensions from different treatments were incubated at 28℃. After an 18-hour incubation period, the centrifuge tubes were removed and mixed using a vortex mixer. Subsequently, the spore germination rate was examined under a microscope. For each treatment including over 100 spores were evaluated, with three replicates per treatment. This experiment was conducted three times. The spore germination rate was calculated using the formula: Spore germination rate = (number of germinated spores/total number of spores) ×100%.

### 2.6 Effects of BL06 cell-free supernatant (CFS) on mycelial morphology and spore germination of *C. fimbriata*

For dual culture assays, two symmetrical holes (5 mm diameter) were made in PDA plates, 2.5 cm from the center. The CFS (50 µL) of strain BL06 was added to each hole, with sterile water as the control. The CFS of strain BL06 was prepared as previously described (Xu et al., 2021). A mycelial plug was placed at the center of the plate. The plates were incubated at 28°C with three replicates for each treatment. This experiment was repeated three times. Mycelial diameter was measured at different time points, and the inhibition rate was calculated using the formula: Inhibition rate (%) = (diameter of the control - diameter of the treatment)/(diameter of the control)× 100.

Additionally, the effects of the broth (bacterial suspension), CFS, and resuspension (the bacterial suspension was subjected to centrifugation to discard the supernatant and then resuspended in sterile water) on mycelial morphology were evaluated as described in Method 2.4. Briefly, after three days of exposure to the treatments, *C. fimbriata* mycelia were sampled and observed under a microscope for changes in morphology, including swelling, deformation, and abnormal growth patterns. Mycelia treated with PDA alone were used as the control group.

To evaluate the impact on spore germination, the broth, CFS, and resuspension were tested according to the protocol outlined in Method 2.5. The bacterial suspension and its resuspension were maintained at 1×10^6^ CFU/mL.

### 2.7 Antifungal susceptibility testing

According to previous research, CFS’s minimum inhibitory concentration (MIC) was measured for *C. fimbriata* using a dilution method with appropriate modifications (Li et al., 2021). CFS was serially two-fold diluted in a 96-well microplate, starting with 180 μL of CFS, and the volume was halved in subsequent wells. The plates were incubated at 28°C for 7 days. After incubation, 0.07 g/L resazurin was added to all wells and incubated for an additional 4 hours. The MIC was determined as the lowest concentration of CFS that prevented the color change from blue (oxidized) to pink (reduced), indicating no fungal growth.

### 2.8 Transcriptome analysis of response to CFS of *C. fimbriata*

The *C. fimbriata* spore suspension (1 × 10^6^ spores/mL) was added to the liquid medium (PDA: CFS=1:1) and cultured for 0, 6, 24, and 48 h at 28°C, 180rpm. 0 h was designated CK, CFS treated 6 h as CFT1, 24 h as CFT2, and 48 h as CFT3. Each treatment was repeated three times. Then, the spores were collected by centrifugation at 12000 rpm for 20 min at 4°C and were washed thrice with sterile water. The *C. fimbriata* spores collected at different time points were sent to Suzhou Panomike Biomedicine Technology Co., Ltd. for total RNA extraction, library construction and sequencing. Quality control was performed on the raw data to obtain high-quality data. Then, the data was mapped to the sweet potato genome (GCA_000389695.3) using HISAT2 (v2.0.5) and the resulting data was spliced into transcripts. Gene expression levels were quantified using fragments per kilobase per million (FPKM). Differential expression analysis (DEGS) was conducted with a significance threshold of P-value< 0.05 and | log_2_ (fold-change) | ≥ 1. Enrichment analyses for Gene Ontology (GO) and Kyoto Encyclopedia of Genes and Genomes (KEGG) were performed on the differentially expressed genes using cluster Profiler (v3.8.1) software.

### 2.9 qRT-PCR analysis

A quantitative real-time polymerase chain reaction (qRT-PCR) analysis was conducted to validate the RNA-seq data. The qRT-PCR reaction (total volume 20 µL) contained the following components: 10 µL SYBR mix, 0.4 µL forward primer, 0.4 µL reverse primer, and 5.6 µL double-distilled water. The PCR cycling conditions were as follows: initial denaturation at 95°C for 3 minutes, followed by 30 cycles of denaturation at 95°C for 15 seconds, annealing at 58°C for 20 seconds, and extension at 72°C for 30 seconds. Gene expression levels were calculated using the 2^−ΔΔCt^ method based on the Ct values obtained from the qPCR reaction. Actin-related protein 3 (*arp3*) was used as the reference gene. The experiment was repeated three times, and the primers used are listed in **Supplementary Table S1**.

### 2.10 Statistical analysis

All statistical analyses were performed using GraphPad Prism 8.0. For comparisons among multiple groups, a one-way analysis of variance (ANOVA) was applied. For comparisons between two groups, a two-tailed unpaired t-test was used. Statistical significance was assessed at p< 0.05. For two-group comparisons, significance levels were indicated as follows: *P< 0.05, **P< 0.01, and ***P< 0.001. For multiple group comparisons, significant differences were denoted using different lowercase letters (P< 0.05). Results are expressed as the mean ± standard deviation (SD). Each experiment was conducted with three biological replicates and repeated three times.

## 3 Results

### 3.1 Antifungal activity of strainBL06 against *C. fimbriata*

**Figure 1:**
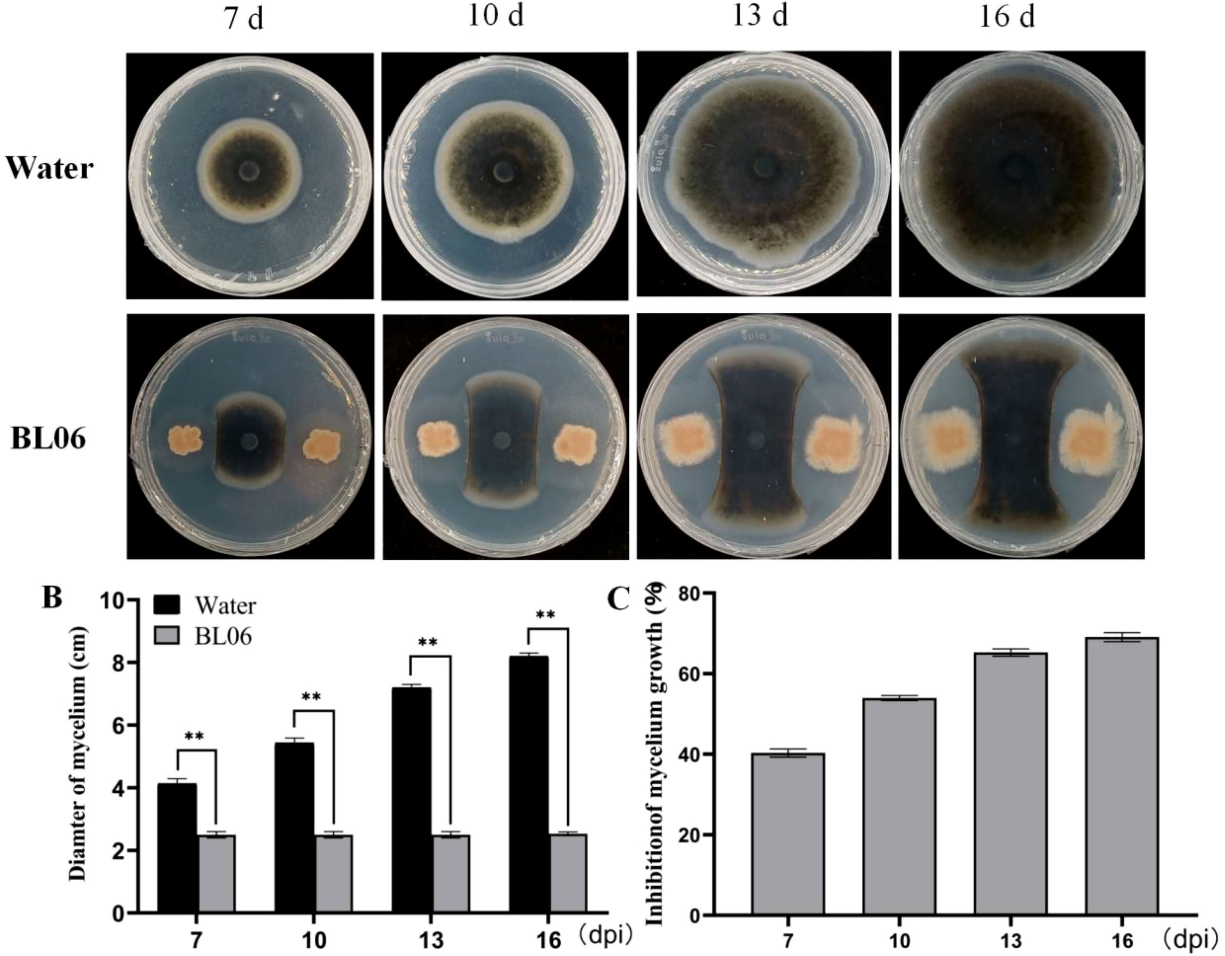
Antifungal effects of strain BL06 against *C. fimbriata in vitro*. **(A)** Comparison of *C. fimbriata* mycelial growth on PDA medium in the absence and presence of strain BL06, sterile water as a control. **(B)** Diameter of *C. fimbriata* mycelial growth **(D)** Visual representation of the inhibition rate of strain BL06 on *C. fimbriata* mycelial diameter at 7, 10, 13, and 16 days post-inoculation (dpi). Significance levels: **P< 0.01.

The antagonistic effect of BL06 on *C. fimbriata* was investigated. In the plate antagonism experiment, the radial expansion of mycelium towards BL06 was significantly suppressed compared to the control group, demonstrating BL06’s consistent and potent antifungal activity (**Figures 1A, B**). After 7, 10, 13, and 16 days of incubation, the inhibition rates of mycelial growth, calculated by comparing treated and control plates, were found to be 39.53%, 53.57%, 64.38%, and 69.11%, respectively (**Figure 1C**). These results indicate that BL06 can effectively inhibit mycelium growth of *C. fimbriata in vitro*.

### 3.2 BL06 inhibited infection of sweet potato by *C. fimbriata*

**Figure 2:**
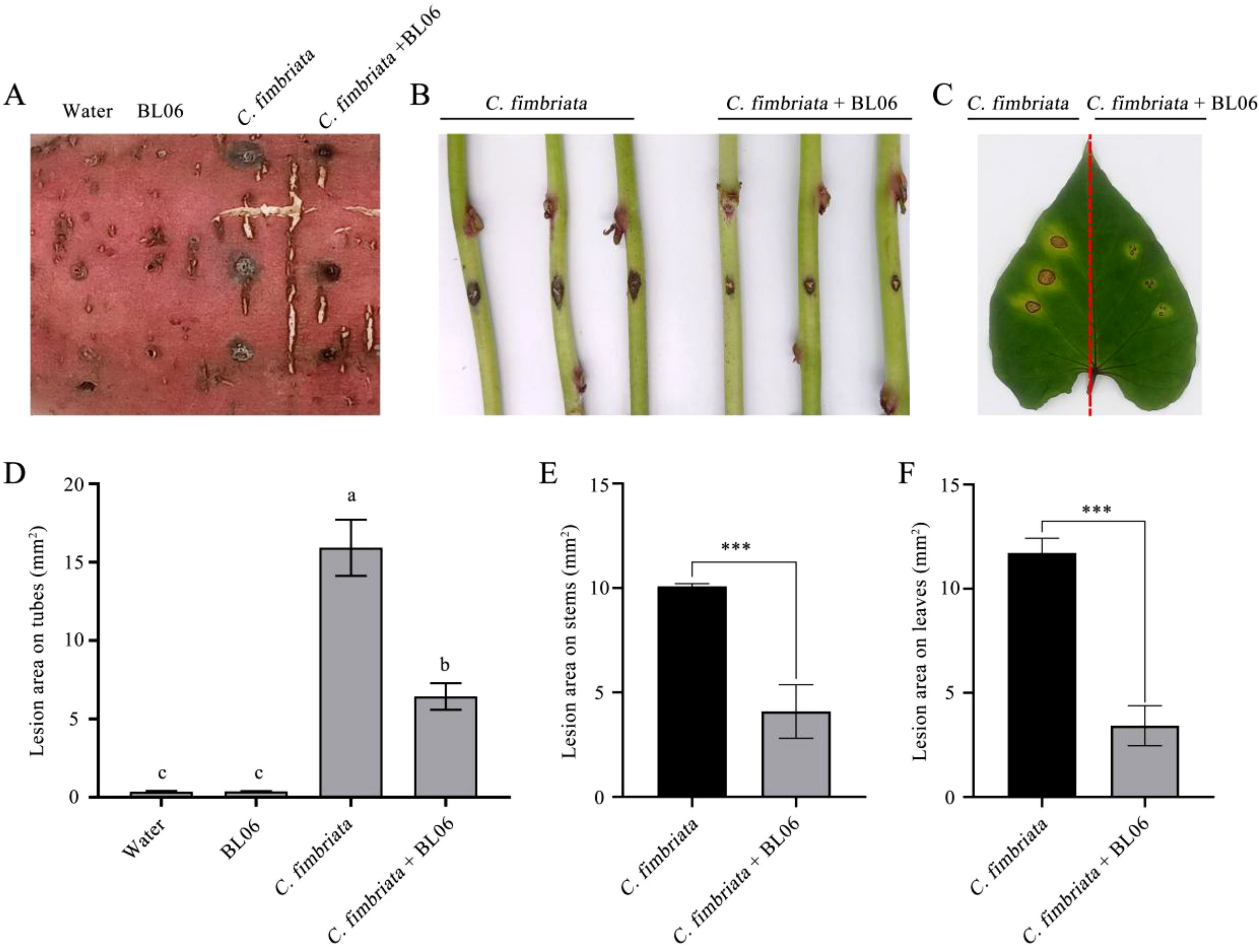
Effect of strain BL06 against *C. fmbriata* on storage roots, the detached stems and leaves of sweet potato (*Ipomoea batatas* “Xu18”). **(A)** After 10 days of disease incidence on storage roots, the roots were pretreated with BL06 or sterile water (control) for 1 day, and then the spores of *C. fmbriata* were inoculated. The incidence area was counted 10 days later (scale bar=1 cm). **(B)** Disease incidence of the stems of sweet potato plants at 36 h. The stems of sweet potato plants were pre-treated with sterile water (control) (control; left) and BL06 (right) for one day after co-incubation with the spores of *C. fmbriata*. **(C)** Disease incidence in the leaves of sweet potato plants at 36 (h) The leaves were pre-treated with water (control; left) and BL06 (right) at 36 h after co-incubation with the spores of *C. fmbriata.* **(D, E, F)** were areas of the lesion region on storage roots, the detached stems and leaves of sweet potato, as evaluated by ImageJ analysis. The values shown above are averages of at least three independent experiments. The error bar represents the standard error of the mean value (SEM). Different lowercase letters (e.g., “a” and “b”) indicated significant differences (n ≥ 3, P ≤ 0.05), and Asterisks indicated statistical significance by one-way ANOVA and LSD test (P< 0.05). Significance levels: ***P< 0.001.

To estimate the biocontrol efficacy of BL06 against sweet potato black rot, the tubers, stems and leaves of sweet potato were inoculated with the strain BL06 suspension and *C. fimbriata* spores. The results demonstrated that leaves and stems treated with water exhibited symptoms of black rot disease. After 48 hours of inoculation, dark brown spots appeared on the upper surface of the leaves and stems, accompanied by yellowing in the surrounding area (**Figures 2B, C**). Notably, the BL06 pretreatment group displayed significantly smaller lesions than the water treatment group (**Figure 2A**). Furthermore, the tubers subjected to BL06 treatment exhibited a significant reduction in disease area compared to those treated with water (**Figures 2D, E**). These findings highlight the effective control efficacy of BL06 on sweet potato black rot.

### 3.3 Strain BL06 altered the morphology and activity of *C. fimbriata* mycelia

**Figure 3:**
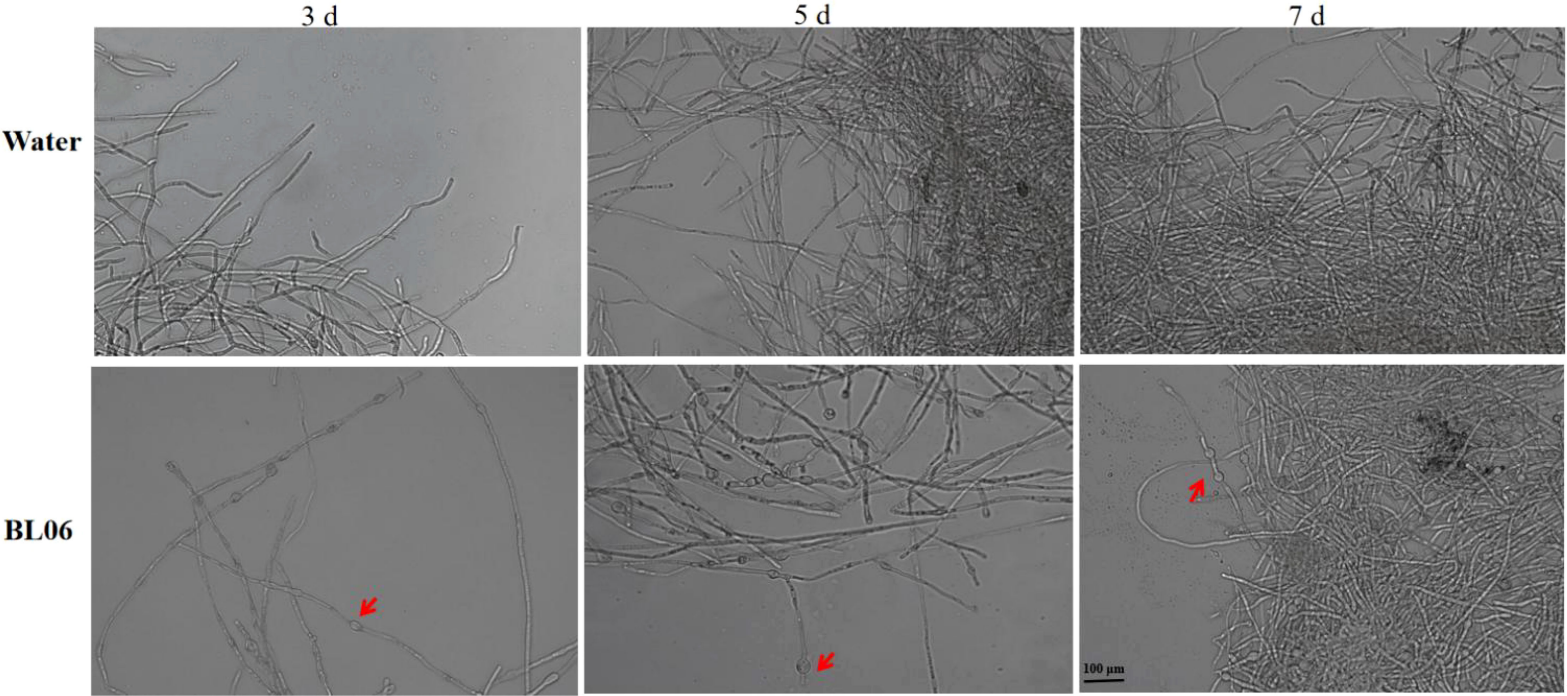
Effect of strain BL06 on mycelial morphology of *C. fmbriata* at different times. The mycelium of *C. fmbriata* was treated with sterile water (control) and the BL06 concentration was 1 × 10^6^ CFU/ml. The mean and standard deviation were calculated using three bioduplicates. Scale bar was 100 μm.

To assess the impact of BL06 on the mycelial morphology of *C. fimbriata*. We conducted an observation of mycelial morphology. As depicted in **Figure 3**, the control group exhibited full and regular mycelia with a smooth and healthy surface. However, swelling and deformation were observed in the mycelia on days 3, 5, and 7 after BL06 treatment, indicating a significant alteration in mycelial morphology and its impact on trophic growth. Furthermore, staining with Neutral red and Evans blue revealed that BL06 could affect the activity of *C. fimbriata* mycelia (**Supplementary Figure S1**).

### 3.4 BL06 significantly inhibited the spore production and germination of *C. fimbriata*

**Figure 4:**
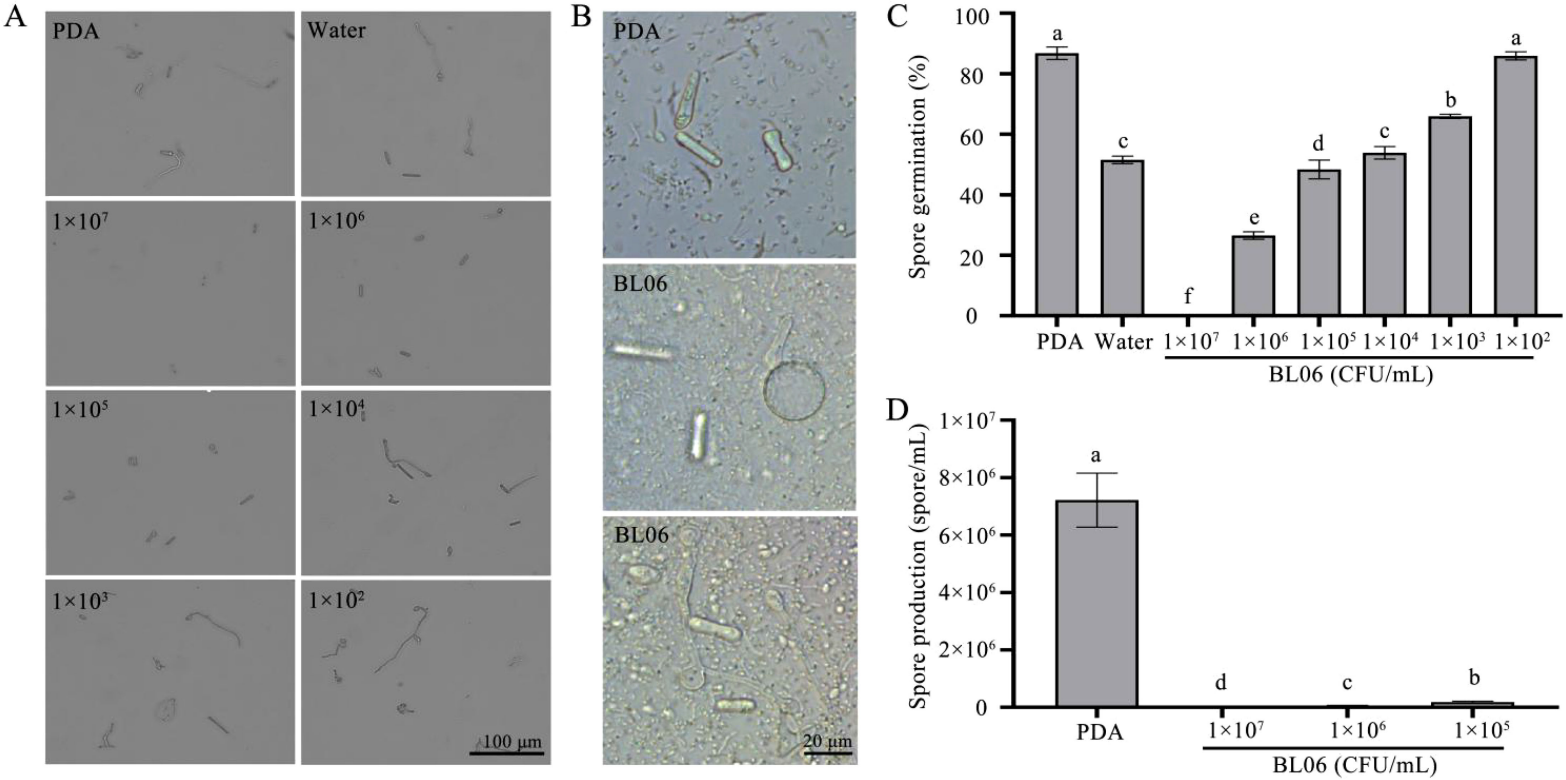
Inhibition of *C. fimbriata* spore germination and production by strain BL06. Spores of *C. fimbriata* were treated with sterile water, PDA and BL06 at the concentrations indicated. **(A)** The germination of (*C*) *fimbriata* spores was observed for 18 h under a microscope. Scale bar was 100 μm. **(B)** Effect of BL06 on mycelium morphology and germination of spore. **(C)** Germination of the sterile water, PDA and BL06-treated spores at 18 h. **(D)** The formation of spores were observed under a microscope. Bar = 100 μm. Data from three biological replicates were used to calculate the mean and standard deviation. Error bars represent the standard error of the mean of three independent replicates. Lowercase letters indicated significant differences (n ≥ 3, P ≤ 0.05).

Normal mycelia were treated with varying concentrations of BL06, resulting in a significant reduction in spore production compared to the control group. The number of spores produced was respectively 7.22 × 10^6^ spores/mL (PDA), 1.83 × 10^5^ spores/mL (1 × 10^5^ CFU/mL), 6.17 × 10^4^ spores/mL (1 × 10^6^ CFU/mL), 0 spores/mL (1 × 10^7^ CFU/mL) (**Figure 4D**). These results suggest that the inhibitory effect of BL06 is dose-dependent and related to its concentration. In addition, the inhibitory effect of BL06 on spore germination was also concentration-dependent (**Figure 4A**). The spores were treated for 18 hpi with PDA, sterilized water, and varying concentrations of BL06. The germination rates were as follows: PDA (87.92%), 1 × 10^2^ CFU/mL (85.93%), 1 × 10^3^ CFU/mL (65.93%), 1 × 10^4^ CFU/mL (53.88%), 1 × 10^5^ CFU/mL (48.38%), 1 × 10^6^ CFU/mL (26.56%), and 1 × 10^7^ CFU/mL (0%) (**Figure 4C**). Corresponding inhibition rates were 1.01%, 24.03%, 37.96%, 44.31%, 69.37%, and 100%, respectively. Furthermore, low concentrations of BL06 caused swelling and deformation among germinated mycelium (**Figure 4B**).

### 3.5 BL06 CFS is the primary functional substance of antifungal activity

**Figure 5:**
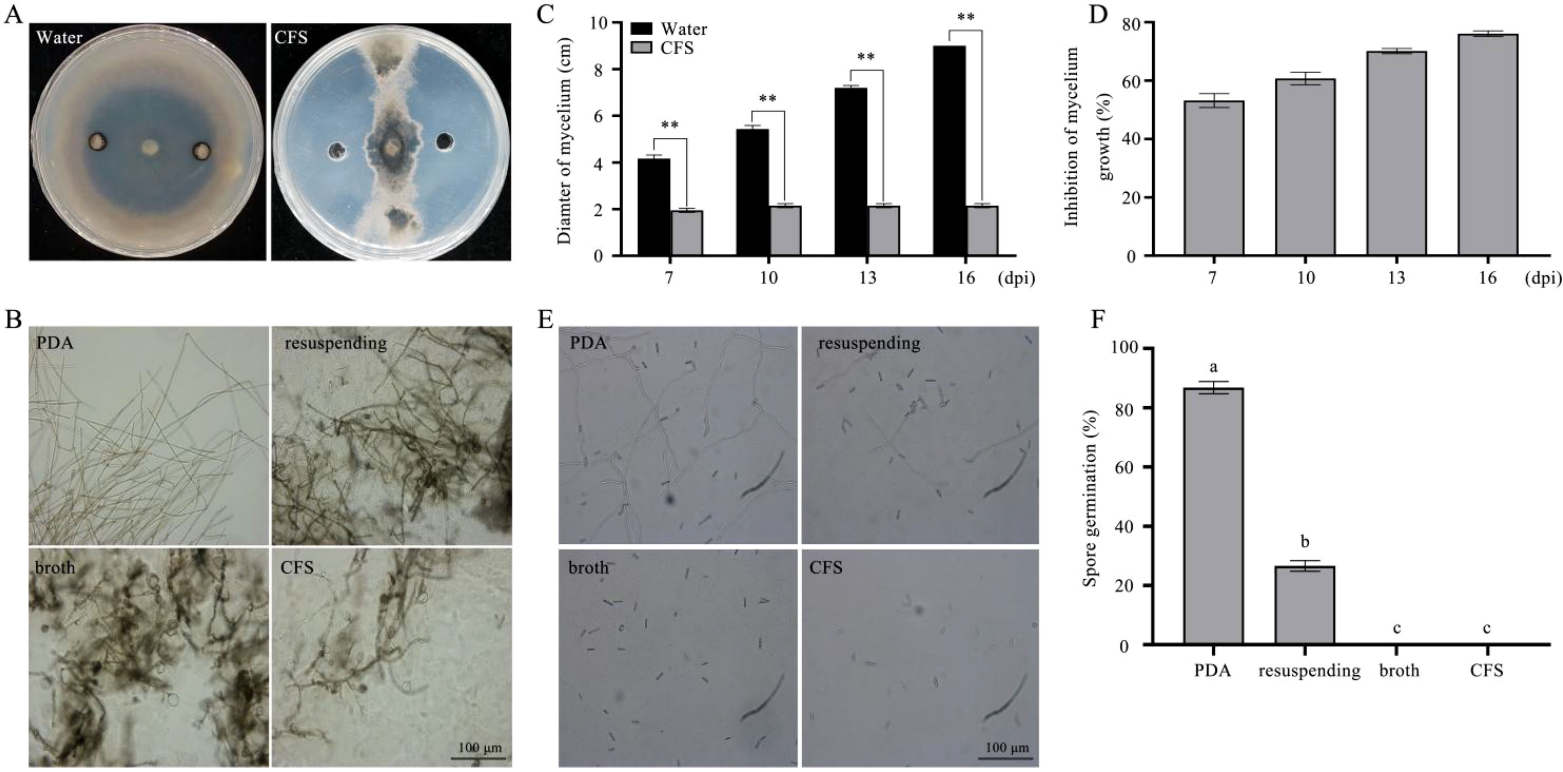
Effect of three components each of BL06 on mycelial morphology and spore germination of (*C*) *fimbriata*. **(A)** The plate antagonism test of the CFS against *C. fimbriata*. sterile water as control. **(B)** The morphology of mycelium was observed under a microscope at 3 d, 5 d, respectively. Scale bar indicated 100 μm. Mycelium was treated with CFS and sterile water. **(C, D)** The CFS significantly inhibited *C. fimbriata* mycelial growth in 7, 10, 13, 16 days post-inoculation (dpi), respectively. **(E)** The germination of (*C. fimbriata* spores was observed at 18 h under a microscope. **(F)** Effect of the CFS on the *C. fimbriata* spore germination rate. Asterisk denote statistical significance (P< 0.05) based on one-way analysis of variance followed by LSD test. Significance levels: **P< 0.01. Different lowercase letters indicated significant differences (P ≤ 0.05).

To investigate the antifungal activity associated with the CFS of strain BL06, we evaluated its effects on the growth and spore germination of *C. fimbriata*. The results showed that the CFS significantly suppressed *C. fimbriata* mycelial growth in PDA medium, with an inhibition rate of 76.11% (**Figures 5A, C, D**), which was notably higher than the effect of the BL06 bacterial suspension (**Figure 1**). Furthermore, exposure to the broth, CFS, and the BL06 bacterial resuspension induced swelling and deformation of *C. fimbriata* mycelia, with the CFS showing the most pronounced effect (**Figure 5B**). The CFS also significantly inhibited spore germination of *C. fimbriata* (**Figures 5E, F**). The inhibition rates of broth, CFS, and the BL06 bacterial resuspension on spore germination were 100%, 100%, and 73.37%, respectively. These results suggest that the CFS contains key components responsible for the antifungal activity of BL06. Furthermore, a 22.5% concentration of CFS was shown to effectively suppress spore germination (**Supplementary Figure S2**).

### 3.6 Overall transcriptional response of *C. fimbriata* to the CFS of strain BL06

**Figure 6:**
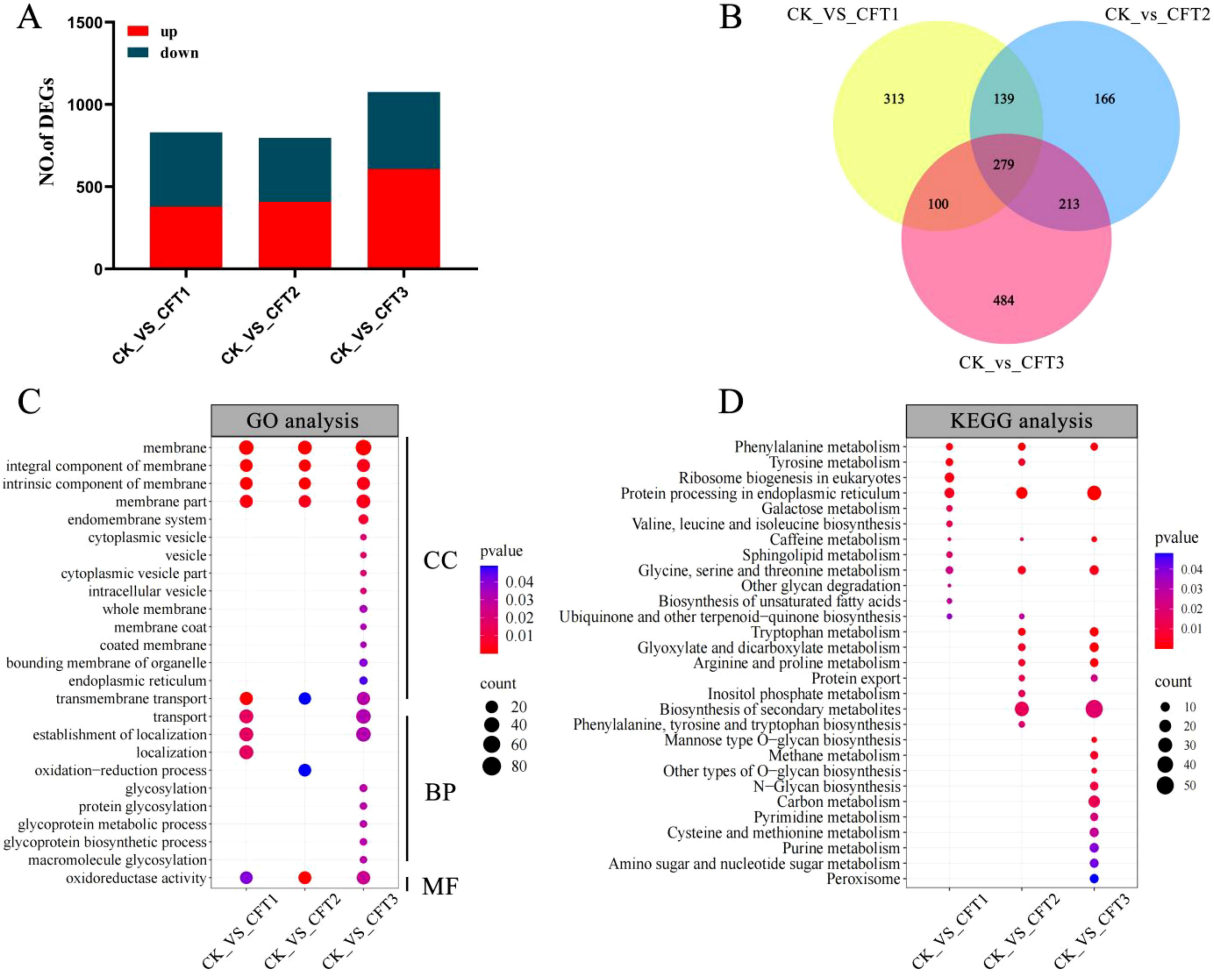
Differentially expressed gene (DEG) analysis of *C. fimbriata* after the CFS treatment. **(A)** Number of upregulated and downregulated DEGs after the CFS treatment. **(B)** Venn diagram of DEGs among the three different treated groups. **(C)** GO pathways significantly enriched (P< 0.05) with DEGs after treated by the CFS. **(D)** KEGG pathways significantly enriched (P< 0.05) with DEGs after treated by the CFS. *C. fimbriata* treated with the CFS for 0 h as CK, 6 h as CFT1, 24 h as CFT2, and 48 h as CFT3.

To further explore the possible antifungal mechanism of BL06 on *C. fimbriata*, we treated *C. fimbriata* spore with the CFS of BL06 and performed transcriptome analysis. After filtering out low-quality data and retaining the high-quality data, a total of 78.68 Gb was obtained. The GC content of all samples was more than 50%, the percentage of Q30 bases was at least 91.45%, and The localization degree with reference genome was more than 91% (**Supplementary Table S2**). The analysis of sequencing data and reproducibility evaluation indicated that the transcriptome data were high quality and appropriate for further analysis (**Supplementary Figure S3**). The selection criteria of DEGs were FDR< 0.01 and∣log_2_(fold-change)∣>1. The results showed that compared with the control group, there were 831 (379 up-regulated and 452 down-regulated), 797 (408 up-regulated and 389 down-regulated) and 1076 (607 up-regulated and 469 down-regulated) differential genes were identified in groups of CFT1, CFT2 and CFT3, respectively (**Figure 6A**). The Venn diagram shows the endemic and shared genes found in each comparison (**Figure 6B**).

Gene ontology (GO) analysis can explain the function of change of DEGs, including molecular function, cellular component, and biological process. GO analysis indicates that the membrane, integral component of membrane, intrinsic component of membrane, membrane part, and oxidoreductase activity were significantly enriched in groups of CFT1, CFT2 and CFT3 (**Figure 6C**). The DEGs were annotated into 29 Kyoto Encyclopedia of Genes and Genomes (KEGG) pathways. In the 6-h treated group, the main enriched KEGG pathways were related to protein processing in endoplasmic reticulum, ribosome biogenesis in eukaryotes, phenylalanine metabolism and tyrosine metabolism. In the 24-h treated group, the most enriched pathways were biosynthesis of secondary metabolites, protein processing in endoplasmic reticulum, phenylalanine metabolism and tryptophan metabolism. In the 48-h treated group, the main pathways were biosynthesis of secondary metabolites, protein processing in endoplasmic reticulum, tryptophan metabolism, arginine and proline metabolism and glyoxylate and dicarboxylate metabolism. Interestingly, KEGG enrichment analysis showed that protein processing in endoplasmic reticulum and phenylalanine were both significantly enriched at 6 h, 24 h and 48 h (**Figure 6D**).

### 3.7 Comparative analysis of DEGs of *C. fimbriata*

**Figure 7:**
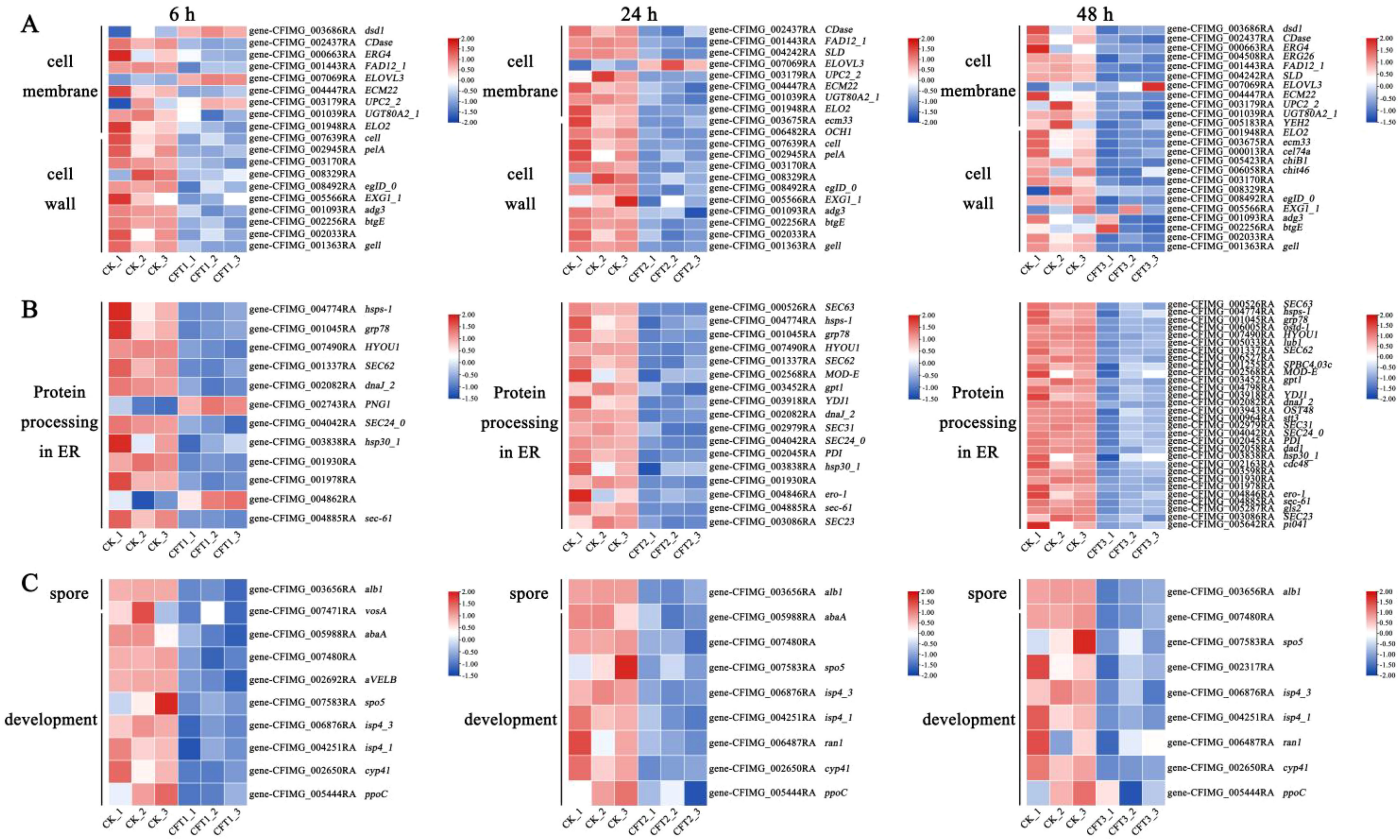
Heatmaps showing relative expression for selected DEGs at 6 h, 24 h and 48 h by the CFS treatment. The log_2_ fold change was colored and standardized. Colors indicate DEGs in the CFS-treated cells versus the control. Red, upregulated; white, not differentially expressed; blue, downregulated. **(A–C)** Shown are DEGs involved in **(A)** cell membrane and cell wall. **(B)** protein processing in endoplasmic reticulum (ER). **(C)** spore development.

The CFS affected the genes related to the cell wall and membrane after treatment of spores. The cell membrane is a crucial part of cell structure and plays an essential role in cellular activities (Horn and Jaiswal, 2019; Peng and Chen, 2024; Malinsky and Opekarová, 2016). In the present study, the genes *YEH2*, *EMC22*, *ERG4* and *erg26* involved in ergosterol biosynthesis were down-regulated at 6 h, 24 h and 48 h after CFS treatment. The plasma membrane component related-gene *UGT80A2_1* and cell membrane fatty acid biosynthesis-related genes *dsd1*, *CDase*, *FAD12_1*, *ELO2*, *SLD* and *CFIMG_005533RA* were also down-regulated at all three treatment time points (**Figure 7A**). The fungal cell wall is a vital cell barrier that plays an important role in the process of fungal growth, maintenance of cell morphology and adaptation to the environment (Geoghegan et al., 2017; Gow et al., 2017);. The genes associated with cell wall biogenesis (*OCH1*, *cel74a*, *pelA*, *chiB1*, *chit46*, *eglD_0*, *EXG1_1*, *adg3*, *btgE*, and *CFIMG_002033RA*) are present in the CFS and their expression was also down-regulated at all three time points after the CFS treatment.

Transcriptome analysis showed that the CFS could significantly inhibit the expression of genes related to spore germination, spore growth and spore development. Conidial pigment polyketide synthase (*alb1*), sexual differentiation process proteinisp4 (*isp4_1*), sporulation-specific protein 5 (*spo5*), linoleate 10R-lipoxygenase (*ppoC*), were downregulated 3.54-fold, 1.96-fold, 3.27-fold, 1.62-fold and 1.68-fold, respectively, at 6 h. These genes were downregulated 3.67-fold, 1.89-fold, 3.38-fold, 1.53-fold and 3.05-fold, respectively, at 24 h. These genes were also downregulated 3.45-fold, 1.13-fold, 2.47-fold, 3.05-fold and 1.68-fold, respectively, after 48 h of inoculation. Spore development regulator vosA (*vosA*), and velvet complex subunit B were downregulated 1.90-fold and 1.14-fold after treatment for 6 h. Sexual differentiation process protein *isp4* and negative regulator of sexual conjugation and meiosis (*ran1*) were 1.23-fold and 1.03-fold, after treatment for 24 h (**Figure 7C**).

The endoplasmic reticulum is an important organelle for protein folding and processing (Askew, 2014; Krishnan and Askew, 2014). The expression of *C. fimbriata* protein processing in endoplasmic reticulum genes was down-regulated after the CFS treatment. These genes included protein transport protein genes (*SEC23*, *SEC24_0*, *SEC31*, *sec-61*, *SEC62*, *SEC63*), molecular chaperon-related genes (*PDI*, *dnaJ_2*, *CFIMG_006527RA*), endoplasmic reticulum associated degradation system genes (*hsp30_1*, *hsps-1, MOD-E*), etc (**Figure 7B**).

In this study, we found that the CFS negatively affected the expression of *C. fimbriata* genes related to energy metabolism. These down-regulated genes include members of the ATPase family, transporter ATPases, ATP-dependent helicases, and ATPase subunits, which are involved in various cellular activities (**Supplementary Figure S4A**). The *rad50* gene is involved in DNA double-strand break repair of ATPase, and the expression level of ATPase was down-regulated by 1.14 times at 6 h. The *RAN1_1* gene, which encodes copper transport ATPase was downregulated by 1.76, 1.74 and 1.75 times after treatment for 6, 24 and 48 hours, respectively. The *gacS* gene encoding the sensor protein was downregulated by 1.36, 1.6 and 1.28 times after 6 h, 24 h and 48 h, respectively. In add9ition, 16 genes related to mitochondrial function were also down-regulated after treatment of the spores with CFS (**Supplementary Figure S4B**). qRT-PCR analysis was carried out on six selected genes, showing a consistent expression pattern with RNA-seq results (P< 0.05), confirming the reliability of our RNA-seq data (**Supplementary Figure S5**).

## 4 Discussion

In this study, we analyzed the antagonistic effects and underlying mechanisms of *B. licheniformis* BL06 against *C. fimbriata*. The results demonstrated the strong antifungal activity of BL06 and revealed its multifaceted mechanisms of action through morphological observations, functional assays, and transcriptomic analysis.

The plate antagonism assay showed that BL06 significantly inhibited the growth of mycelia, and the inhibition rate was up to 69.11% (**Figure 1C**). Moreover, the laboratory biocontrol test showed that the strain BL06 proved to be effective in controlling sweet potato black rot in leaf, stem and root through decreasing the lesion area (**Figure 2**). This result was similar to the results of *Pantoea dispersa* RO-18, RO-20, RO-21 and *B. amyloliquefaciens* YTB1407 (Jiang et al., 2019; Wang et al., 2020). After treatment with BL06, the morphology of the mycelia changed, swelling appeared, and mycelial activity decreased (**Figure 3**; **Supplementary Figure S1**). We also investigated the effects of BL06 on the sporulation and spore germination of *C. fimbriata in vitro*. The results showed that BL06 could significantly suppress spore production and spore germination of *C. fimbriata* in dose-dependent (**Figures 4A–D**).

Interestingly, spores treated with the bacterial solution exhibited circular swelling, and the germinated mycelia displayed deformed swelling (**Figure 4B**), a phenomenon not previously reported for other biocontrol bacteria acting on *C. fimbriata*. Treatment with the broth, CFS, and BL06 bacterial resuspension resulted in swelling and deformation of *C. fimbriata* mycelia, with the most significant effect observed in the CFS treatment (**Figure 5B**). These results suggest that BL06’s antifungal components disrupt mycelial structure and inhibit fungal growth. To further identify the antifungal components of BL06, we tested its broth, resuspension, and CFS against *C. fimbriata*. The results revealed that CFS had a stronger inhibitory effect on mycelial growth compared to the bacterial resuspension (**Figures 1A**, **5A**). Additionally, spores treated with CFS and broth did not germinate, whereas the resuspension allowed 26.63% germination (**Figures 5E, F**). These findings align with a similar effect of BL06 on *Magnaporthe oryzae* spore germination (Liu et al., 2021). Taken together, the results suggest that strain BL06 inhibits the growth of *C. fimbriata* primarily through the antifungal components in the CFS, which not only suppress mycelial growth but also prevent spore germination.

Recent studies have revealed that the antimicrobial activity of biocontrol bacteria involves multiple layers, encompassing cellular development, metabolic processes, and genetic information handling (Compant et al., 2005; Saeed et al., 2021; Zhang D. et al., 2022). Transcriptome sequencing can investigate the specific mechanism of the mode of action of BL06 against the *C. fimbriata*. Transcriptome analysis showed that 2704 genes were significantly differentially expressed (**Figure 6A**). The results showed that the CFS had many effects on the gene transcription of the *C. fimbriata*, and also indicated that many biological processes of the *C. fimbriata* were changed due to the CFS. Annotation analysis of DEGs showed that these genes were mainly concentrated in membrane transport, energy metabolism, protein processing in endoplasmic reticulum, phenylalanine metabolism and other pathways.

The cell membrane is an essential target for biological control (Sant et al., 2016). Transcriptome results revealed that the CFS of *B. licheniformis* BL06 suppresses key genes involved in ergosterol and fatty acid biosynthesis, indicating that fungal cell membranes constitute a primary target of its antifungal activity. Fatty acid biosynthesis genes, such as *dsd1*, *CDase*, *FAD12_1*, *ELO2*, *SLD*, and *CFIMG_005533RA*, are critical for maintaining membrane fluidity and structural integrity. The suppression of these genes suggests that BL06 may interfere with lipid biosynthesis, which could compromise membrane integrity and permeability. Notably, *ELO2* is essential for forming lipid rafts, which is crucial for membrane protein function and cellular signaling (Olson et al., 2015). Additionally, the downregulation of ergosterol biosynthesis genes (*ERG6* and *ERG7*) compromises fungal membrane stability. Ergosterol, a key sterol in fungal membranes, ensures membrane fluidity and supports the activity of membrane-bound enzymes (Choy et al., 2023). Its depletion is likely responsible for the observed morphological alterations, such as membrane swelling and deformation (**Figure 3**), ultimately leading to fungal cell death. These findings align with previous observations of similar antifungal mechanisms in *Pseudomonas chlororaphis* subsp. *aureofaciens* SPS-41 (Zhang et al., 2019).

CFS treatment also possibly interferes with cell wall biosynthesis in *C. fimbriata*, resembling the inhibitory effects of volatile organic compounds (VOCs) from WY228 (Liu et al., 2021). This destabilization likely explains the swelling and deformation observed in fungal mycelia and spores. Transcriptomic analysis revealed substantial suppression of genes essential for cell wall biosynthesis, such as *CHS2* (encoding chitin synthase), *GLO1* (encoding glucan synthase), and *OCH1* (involved in N-glycan processing), as well as genes related to cell wall remodeling, including *cel74a* and *pelA*. Chitin and glucans are crucial for maintaining cell wall structure, and their reduced synthesis renders the wall more susceptible to external stress (Gow et al., 2017). The suppression of remodeling genes further disrupts cell wall maintenance, impairing fungal growth and adaptability. These findings align with the observed morphological abnormalities, such as swelling and deformation (**Figure 4B**), and correspond with findings reported for *B. velezensis* strain YB-185 inoculated with *Fusarium pseudograminearum* (Zhang J. et al., 2022). However, BL06’s simultaneous inhibition of genes involved in both biosynthesis and remodeling indicates a more comprehensive and potent antifungal mechanism.

Spores are essential for fungal survival, transmission, and infection (Kong et al., 2023). The CFS treatment significantly downregulated genes involved in spore germination and development, in agreement with prior studies demonstrating that BL06 inhibits mycelial growth, decreases sporulation, and suppresses spore germination. Comparable effects were observed with *B. altitudinis* strain HSSN09 in combating *Fusarium oxysporum* f. sp. *niveum* (Yang et al., 2024). Key sporulation-related genes, such as *alb1* (conidial pigment synthesis), *isp4_1* (sexual differentiation protein), and *spo5* (sporulation-specific protein 5), were markedly downregulated, with this suppression sustained at 6, 24, and 48 hours. For instance, *alb1* expression was downregulated by 3.54-fold, 3.67-fold, and 3.45-fold at these respective time points. Morphological abnormalities in spores, including swelling and deformation (**Figure 4B**), may result from impaired osmotic regulation and cell wall instability. This observation is corroborated by the suppression of *vosA* and *velB*, genes essential for spore viability and structural integrity (Wu et al., 2021). These disruptions impair spore formation and germination capacity, thereby curtailing the pathogenicity and spread of *C. fimbriata*. Comparable effects have been reported for other biocontrol agents, such as *B. amyloliquefaciens* SFB-1 (Gao et al., 2024) and *Paenibacillus polymyxa* strain NX20 (Yu et al., 2023).

The endoplasmic reticulum (ER) is essential for fungal growth, development, and virulence, as it regulates protein folding, transport, and stress responses (Schirawski et al., 2005; Zhou et al., 2016). KEGG pathway analysis revealed a significant enrichment of genes associated with protein processing in the ER (**Figure 6D**). Following BL06 CFS treatment, key ER-related genes, including those involved in molecular chaperoning, protein transport, and the ER-associated degradation (ERAD) system, were markedly suppressed (**Figure 7B**). This finding indicates that the CFS disrupts ER functionality and compromises fungal growth and pathogenicity. Specifically, the CFS selectively downregulated genes related to protein folding (*PDI1*, *EMP47*, *dnaJ_2*), protein transport (*SEC23*, *SEC24_0*, *SEC31*), and the ERAD system (*hsp30_1*, *hsps-1*), resulting in the accumulation of misfolded proteins, induction of ER stress, and diminished fungal viability. This disruption overwhelms the ER’s quality control mechanisms, exacerbating cellular dysfunction and further restricting fungal growth and virulence (Chen et al., 2023; Poothong et al., 2021). While comparable effects have been reported for other biocontrol agents, such as *Bacillus subtilis* H17-16 (Zhang J. et al., 2023), BL06’s CFS demonstrates broader suppression of ER-related pathways, including protein folding, transport, and degradation, underscoring its distinct and highly effective antifungal mechanism.

The CFS treatment suppressed genes involved in the tricarboxylic acid (TCA) cycle, ATPases, mitochondrial genome maintenance, and functional enzymes (**Supplementary Figure S4**). This disruption is likely to impair mitochondrial function and disrupt energy metabolism, consequently inhibiting *C. fimbriata* mycelial growth, sporulation, and spore germination. Key TCA cycle genes, including *IDH1*, *FUM1*, and *MDH1*, were suppressed, leading to reduced ATP production and limited energy availability. The downregulation of mitochondrial genes associated with ATPases and transporters further suggests mitochondrial dysfunction, thereby restricting the energy supply required for essential cellular processes such as osmotic regulation and cell wall maintenance. This mitochondrial disruption likely contributes to the observed morphological abnormalities in mycelia and spores, such as swelling and deformation. These findings are consistent with studies demonstrating that mitochondrial dysfunction compromises fungal growth and pathogenicity (Li et al., 2021). BL06’s CFS integrates TCA cycle disruption and mitochondrial impairment, which represents a multifaceted antifungal mechanism. Additionally, ER dysfunction caused by BL06 could exacerbate mitochondrial dysfunction, as ER-mitochondria interactions play a vital role in sustaining energy homeostasis and cellular stress adaptation (Malhotra and Kaufman, 2011). The combined disruption of these organelles potentiates the antifungal efficacy of BL06, thereby profoundly restricting fungal growth, reproduction, and stress tolerance.

## 5 Conclusion

The *B.licheniformis* strain BL06 can significantly inhibit the *C. fimbriata* growth, sporulation and spore germination *in vitro* and effectively control the black rot disease of sweet potato *in vivo*. Furthermore, we proved that the strain BL06 CFS was the primary functional substance of antifungal. The possible mode of action may include inhibiting the expression of genes related to cell wall integrity, the cell membrane, spore germination, processing in endoplasmic reticulum, and energy metabolism in *C. fimbriata*. In conclusion, our study indicates that strain BL06 is a potential biocontrol agent against sweet potato black rot.

## Data Availability

The data presented in the study are deposited in the FigShare repository, accession number 10.6084/m9.figshare.28440530.
