# Supplementary material for: Antifungal efficacy and mechanisms of Bacillus licheniformis BL06 against Ceratocystis fimbriata
Source: Front Plant Sci. 2025 Feb 28;16:1535296. doi: 10.3389/fpls.2025.1535296 (PMC11906456; doi:10.3389/fpls.2025.1535296)
Supplement: Supplementary file 1 [file DataSheet1.docx]

**Supporting Information**

**Figure. S1.** Determination of mycelial activity of *C. fmbriata* by strain BL06. *C. fmbriata* cells were stained with Evans blue (dead cells stain blue) and Neutral red (viable cells stain red) afer co-cultivation with strain BL06 for 7 days. Scale bars were 20 μm.


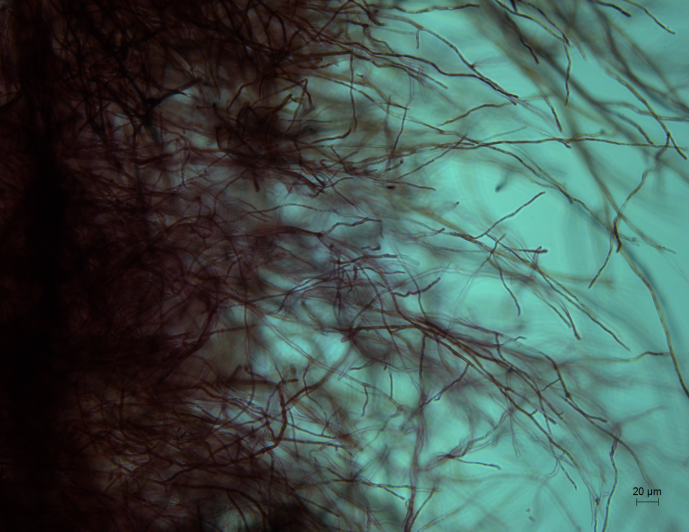

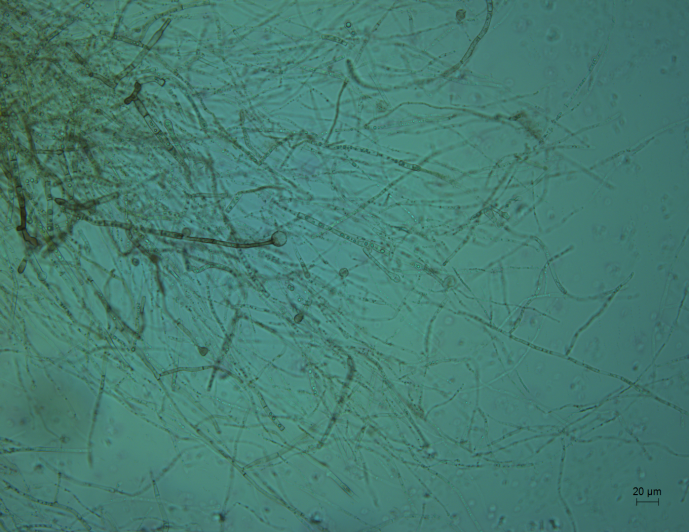

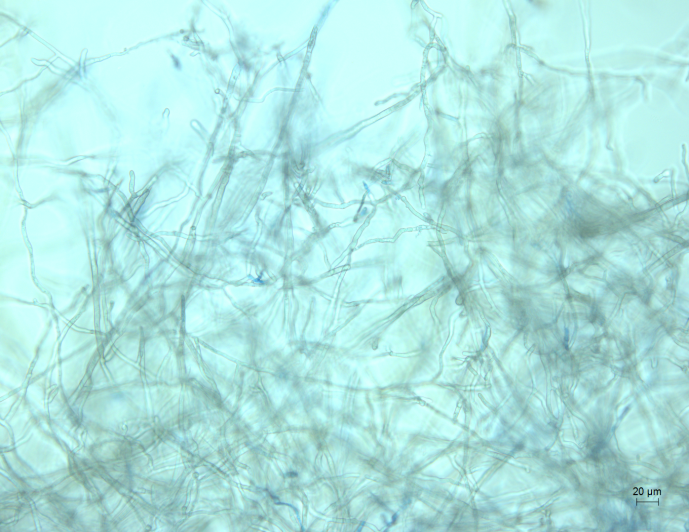

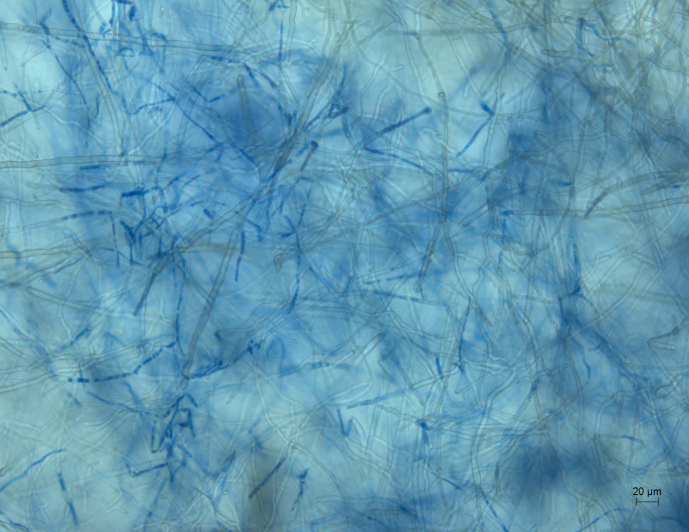


Water

BL06

Evans blue

Neutral red


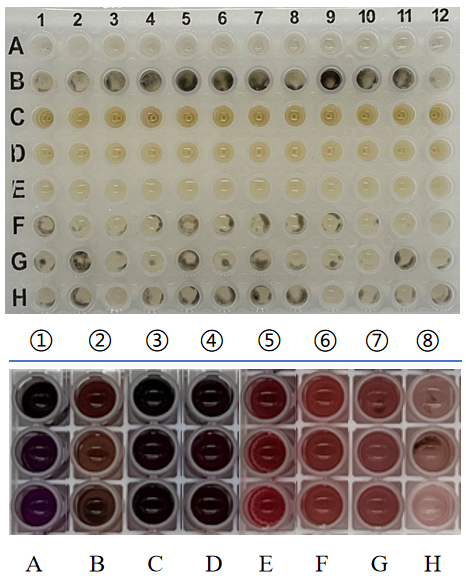


**Figure. S 2.** Minimum inhibitory concentrations (MIC) of cell-free supernatant (CFS) against *Ceratocystis* *fimbriata* were determined using a dilution technique.

Resazurin served as an indicator for clear endpoint determination, where a color shift from blue (oxidized state) to pink (reduced state) signified fungal growth. The MIC was identified as the lowest concentration of the agent that inhibited this color transition.

1. 200 μL PDA. B. 180 μL PDA + 20 μL *C. fimbriata*. C. 180 μL CFS + 20 μL *C. fimbriata*; D. 90 μL PDA + 90 μL CFS + 20 μL *C. fimbriata*. E. 135 μL PDA + 45 μL CFS + 20 μL *C. fimbriata*. F. 157.5 μL PDA + 22.5 μL CFS + 20 μL *C. fimbriata*. G. 168.75 μL PDA + 11.25 μL CFS + 20 μL *C. fimbriata*. H. 174.375 μL PDA + 5.625 μL CFS + 20 μL *C. fimbriata.*


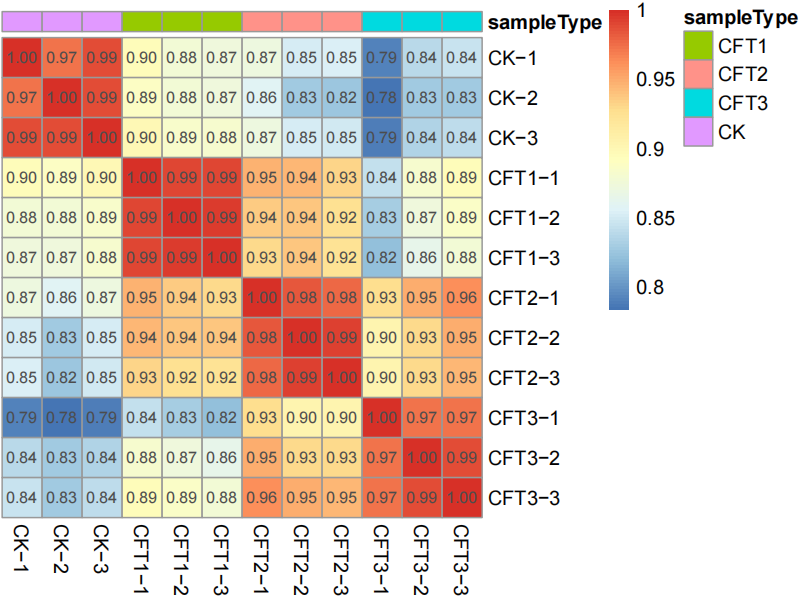


1. of different RNA-seq samples and their PCA analysis.


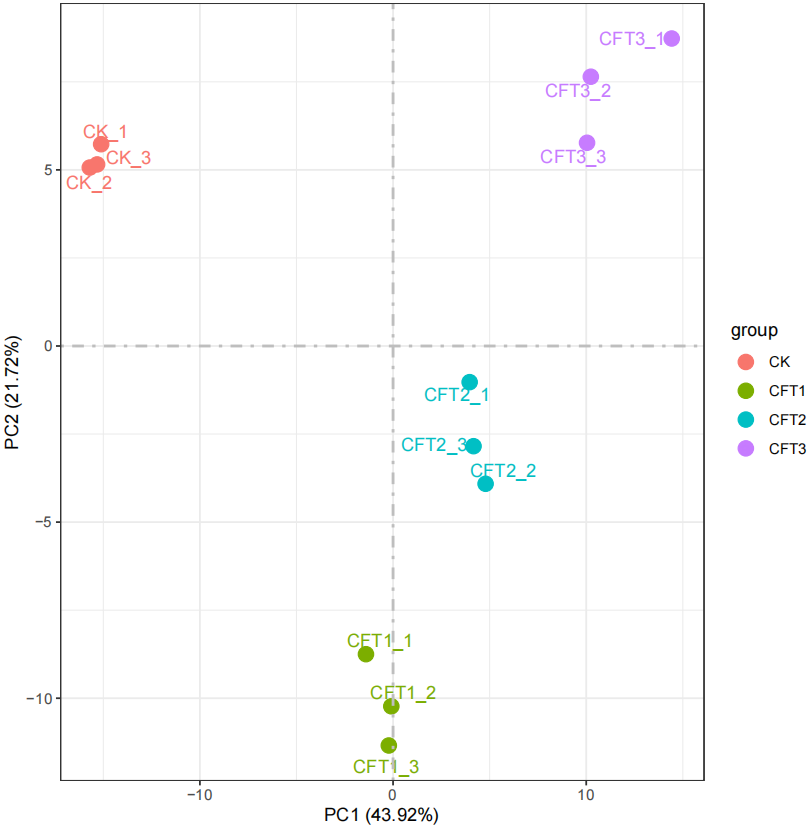


1. the result of PCA analysis.

**Figure. S3.** Heatmap/cluster and correlation analysis (A) of different RNA-seq samples and their PCA analysis (B) result of PCA analysis.


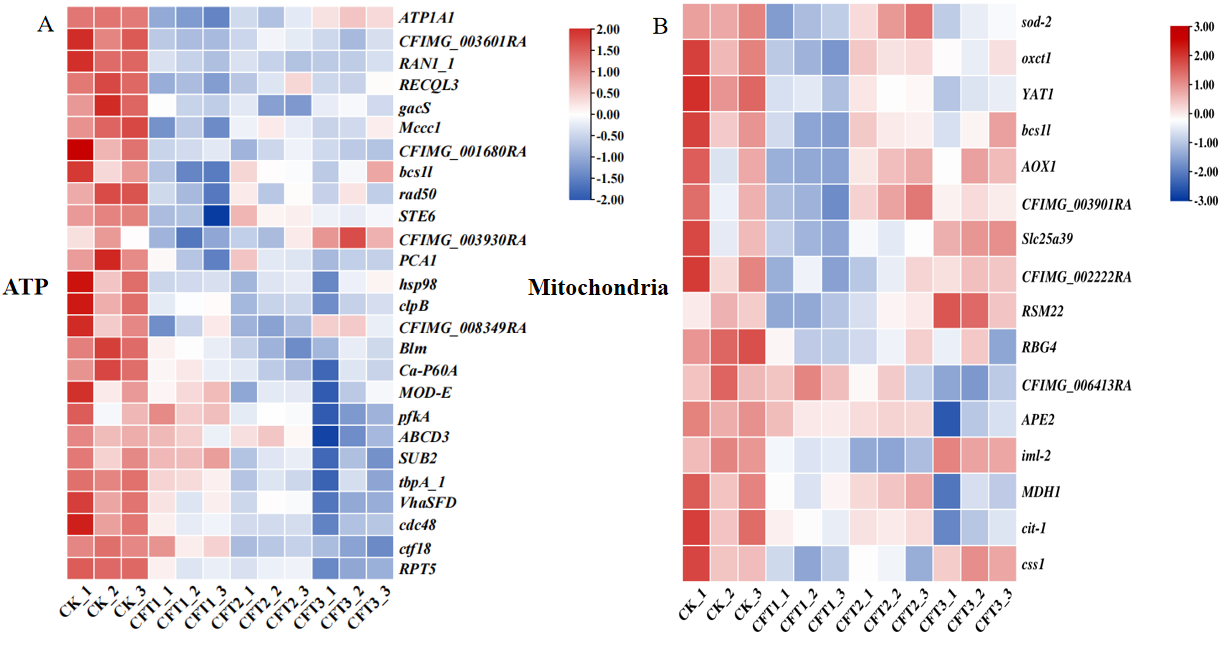


**Figure. S4. Heatmaps showing relative expression for selected DEGs at 6 h, 24 h and 48 h byCFS treatment. Shown are DEGs involved in (A) ATP, (B)mitochondria.**


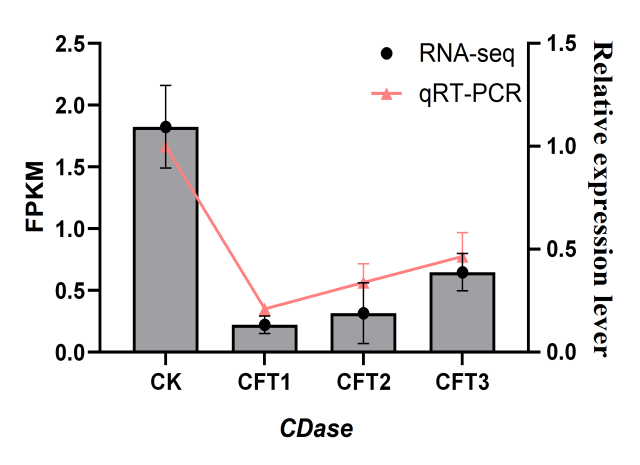


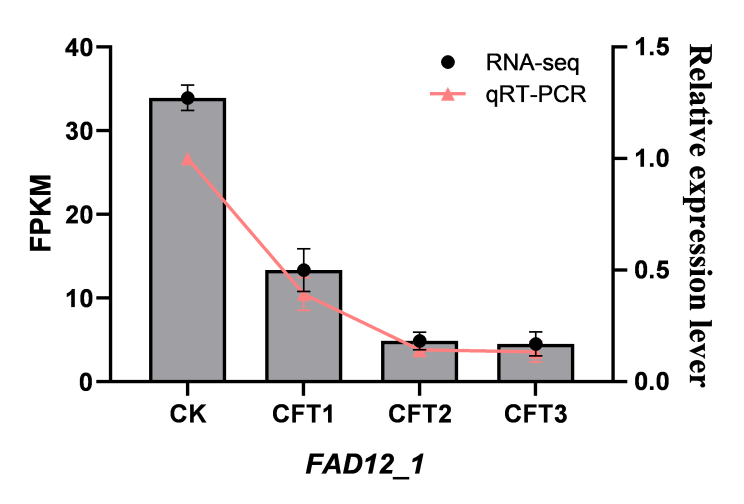

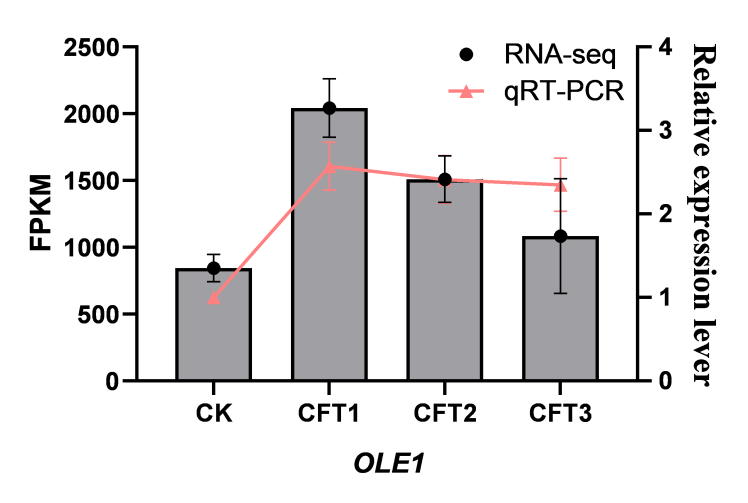

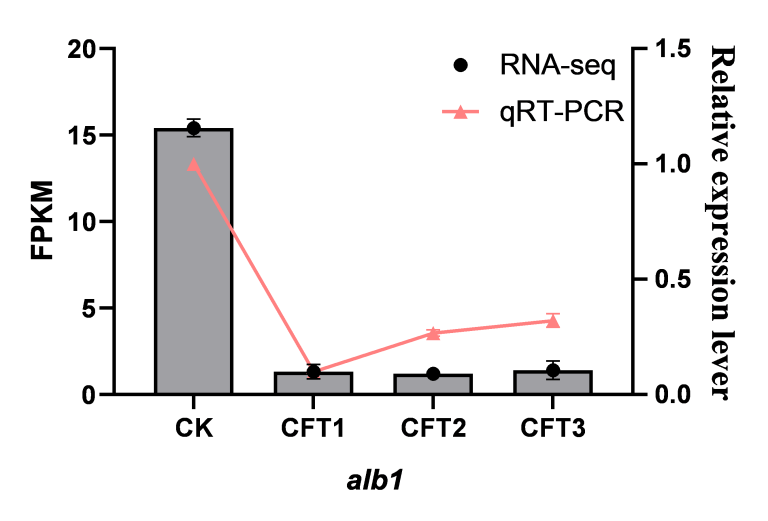

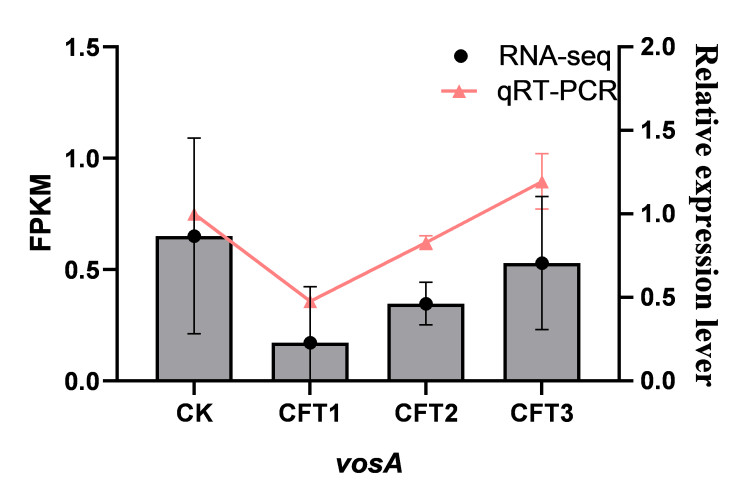

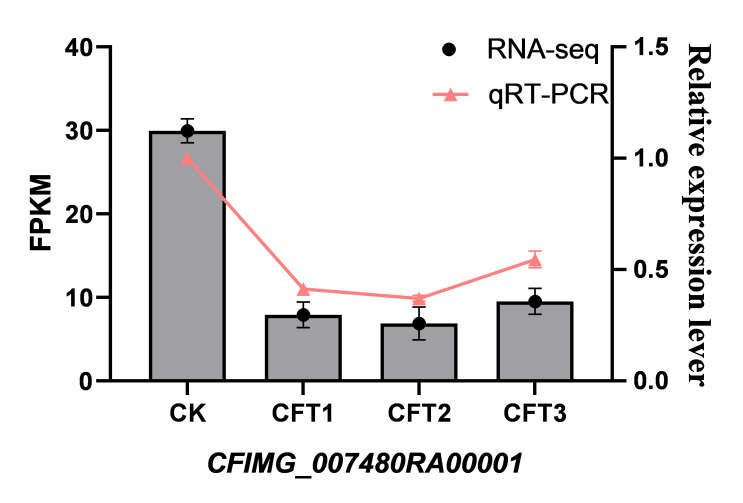


**Figure. S5.** Comparison of the relative gene expression levels of selected genes by

qRT-PCR and RNA-seq in 6 h, 24 h and 48 h cultured *C. fimbriata* cells versus non cultured cells.

**Table. S1** RT-qPCR primer sequences used in this study

| Gene | Gene annotation | Forward (F) and reverse (R) primers |
| --- | --- | --- |
| *β-Actin* | *Actin-related protein 3* | F5’-AGCAGCATGAAGATTAAGGTTGTAGCAC-3’ |
|  |  | R5’-TGGAAAATTAGAAGCACTTCCTGTGAAC-3’ |
| *CDase* | Neutral ceramidase | F5’-CAAGCTCGGTGACGAGTATAAG-3’ |
|  |  | R5*’-*GTGACCTGTGGCAGAAGATAG-3’ |
| *FAD12_1* | Delta(12) fatty acid desaturase | F5’-CCGATAGCTGGTCCTATGTTAAG-3’ |
|  |  | R5*’-*CGACGTGGGTGCCAATAA-3’ |
| *OLE1* | Acyl-CoA desaturase | F5*’-TTCGTAAGGGTCTCCTCTACTC*-3’ |
|  |  | R5*’-* ACAACCACGGCATCTTCAT-3’ |
| *alb1* | Conidial yellow pigment biosynthesis polyketide synthase | F5’-GGTAACATGAAGGAGGGAACTG-3’ |
|  |  | R5*’-* CTCCGCATCACCGTAAAGAA -3’ |
| *vosA* | Velvet factor | F5’-*GGCTCAATGTGTTCGCATATC*-3’ |
|  |  | R5*’-* CTAAAGGAGCGGGTCAAGAA -3’ |
| CFIMG_007480RA00001 | C2H2 finger domain-containing protein | F5’-*CTACACTTCCTCTCCCTCTTCT*-3’ |
|  |  | R5*’-* GCTCATGTTGCCGTTGTTATG -3’ |

**Table. S2** Statistics of transcriptome sequencing data

| **sample** | **library** | **raw_reads** | **raw_bases** | **clean_reads** | **clean_bases** | **Q30** | **GC_pct** | **Total mapped** |
| --- | --- | --- | --- | --- | --- | --- | --- | --- |
| CK_1 | FRAS220297393-1r | 39484188 | 5.92G | 38615030 | 5.79G | 91.86 | 52.62 | 93.21% |
| CK_2 | FRAS220297394-1r | 45742524 | 6.86G | 44940094 | 6.74G | 92.23 | 52.72 | 92.95% |
| CK_3 | FRAS220297395-1r | 42758572 | 6.41G | 41979860 | 6.3G | 91.84 | 52.66 | 94.23% |
| CFT1_1 | FRAS220297396-1r | 43115914 | 6.47G | 42132872 | 6.32G | 91.59 | 52.93 | 93.44% |
| CFT1_2 | FRAS220297397-1r | 47005150 | 7.05G | 46114598 | 6.92G | 91.95 | 52.75 | 94.31% |
| CFT1_3 | FRAS220297398-1r | 46012920 | 6.9G | 45198030 | 6.78G | 91.47 | 52.79 | 94.31% |
| CFT2_1 | FRAS220297399-1r | 48474876 | 7.27G | 47619418 | 7.14G | 92.85 | 52.55 | 93.76% |
| CFT2_2 | FRAS220297400-1r | 46573544 | 6.99G | 45960514 | 6.89G | 93.27 | 52.57 | 94.46% |
| CFT2_3 | FRAS220297401-1r | 45809356 | 6.87G | 44958802 | 6.74G | 93.56 | 52.47 | 94.69% |
| CFT3_1 | FRAS220297402-1a | 41680244 | 6.25G | 40465804 | 6.07G | 92.73 | 51.52 | 94.36% |
| CFT3_2 | FRAS220297403-1r | 46301530 | 6.95G | 45358780 | 6.8G | 92.15 | 52.27 | 93.75% |
| CFT3_3 | FRAS220297404-1r | 42001668 | 6.3G | 41287740 | 6.19G | 91.45 | 52.38 | 93.59% |
